# Chemical Composition and In Vitro Evaluation of Antioxidant and Antiproliferative Effects of Volatile Oils Hydrodistilled from *Onobrychis carduchorum* C.C. Towns., a Kurdish Traditional Plant

**DOI:** 10.3390/plants12163013

**Published:** 2023-08-21

**Authors:** Hawraz Ibrahim M. Amin, Kamaran Abdoulrahman, Azad S. Sadraddin, Heman A. Smail, Zanko Hassan Jawhar, Kovan Dilawer Issa, Chabaco Armijos, Giovanni Vidari

**Affiliations:** 1 Department of Chemistry, College of Science, Salahaddin University-Erbil, Erbil 44001, Iraq; kamaran.abdoulrahman@su.edu.krd (K.A.); heman.smail@su.edu.krd (H.A.S.); 2 Department of Medical Biochemical Analysis, Cihan University-Erbil, Erbil 44001, Iraq; 3 Department of Chemistry, College of Education, Salahaddin University-Erbil, Erbil 44001, Iraq; azad.sadraddin@su.edu.krd; 4 Department of Science, College of Health Science, Lebanese French University, Erbil 44001, Iraq; zanko.jawhar@lfu.edu.krd; 5 Department of Medical Analysis, Faculty of Applied Science, Tishk International University, Erbil 44001, Iraq; kovan.dilawer@tiu.edu.iq (K.D.I.); vidari@unipv.it (G.V.); 6 Departamento de Química, Universidad Técnica Particular de Loja (UTPL), Loja 1101608, Ecuador; cparmijos@utpl.edu.ec; 7 Department of Chemistry, University of Pavia, Via Taramelli 12, 27100 Pavia, Italy

**Keywords:** *Onobrychis carduchorum*, Fabaceae, Kurdish traditional medicine, essential oils, antiradical, antioxidant, antiproliferative effects, β-elemene

## Abstract

The volatile oils hydrodistilled from the aerial parts and roots of *O. carduchorum* C.C Towns. (Fabaceae) have been chemically characterized for the first time. A total of 43 constituents with an abundance >0.03% were identified and quantified in the two oils by GC/MS and GC/FID analyses. They comprise 38 components (98.58%) of the oil isolated from the aerial parts (OCA) and 34 components (93.33%) of the oil from the roots (OCR). Six constituents, α-pinene (23.11 ± 0.1%), β-elemene (17.33 ± 0.1%), 1,8-cineole (12.15 ± 0.2%), furfural (7.91 ± 0.1%), terpineol-4-ol (6.32 ± 0.2%), and limonene (4.13 ± 0.1%), accounted for about 75% of the total OCA oil. On the other hand, 1,8-cineole (15.79 ± 0.1%), furfural (10.44 ± 0.1%), β-elemene (10.14 ± 0.2%), α-terpineol (7.74 ± 0.1%), linalool (7.45 ± 0.1%), and α-pinene (4.76 ± 0.1%) made up about 60% of the OCR oil. The IC_50_ values of the scavenging activities of the OCA and OCR oils towards the DPPH radical and H_2_O_2_ were 79.8 ± 0.5 and 153.3 ± 0.6 μg/mL and 394.09 ± 0.2 and 311.67 ± 0.4 μg/mL, respectively. In addition, in the MTS assay, the OCA and OCR oils showed significant antiproliferative effects against T47D, MDA-MB-453, BG-1, and A549 human cancer cells that were more powerful than those against two normal human cell lines, HEK-293 and HFF-1. The abundant presence of β-elemene as an antiproliferative component of the two oils suggested the existence of a new chemotype of *O. carduchorum*.

## 1. Introduction

Only a few ethnobotanical and phytochemical investigations have been conducted so far on plants growing in the Kurdistan Region of Iraq. Thus, most uses of local traditional plants are not sustained by scientific evidence [1]. The plant *Onobrychis carduchorum* C.C. Towns. (OC) is widely distributed in Iran, Iraq, Syria, and Turkey [2,3,4,5]. In Iraq, it grows mainly on Halgurd Mountain in the Kurdistan region [6]. It is commonly used as an anti-inflammatory and chronic wound healing remedy by Kurds living in a few villages located in the northern region of Choman-Erbil. In medicinal applications, shredded OC leaves are put on skin lesions. In addition, injured skin is cleaned and treated with an infusion prepared by boiling an aqueous suspension of aerial parts for about fifteen minutes. *O. carduchorum*, together with *O. altissima* Grossh., is also mentioned in a detailed survey of medicinal plants used in the Çatak region of Turkey, where the two plants are traditionally used to cure hemorrhages, wounds, and cuts [7]. The morphological, taxonomical, genetic, and ecogeographical characteristics of *O. carduchorum* are well described in the literature [8]. Thus, *O. carduchorum* is easily distinguished from other species belonging to the *Onobrychis* sect. In *Onobrychis*, the length of the keels are considerably shorter than standards [4,5,8].

The genus *Onobrychis* Mill. is the largest in the tribe Hedysareae (family Fabaceae) [8,9,10], which includes more than 170 annual or perennial species. Moreover, more taxa are continuously added to the genus, while others are revised, using both morphological and molecular evidence using the nuclear ribosomal DNA internal transcribed spacer (nrDNA ITS) [10]. Wild *Onobrychis* species grow mainly in Southwest Asia and the Mediterranean region, in different environments. Iran and Anatolia are biodiversity centers for the genus [10], while fourteen species grow in Iraq [3]. A few species, for example, *O. viciifolia* (sainfoin), are also cultivated as important fodder or ornamental plants [11].

Only a few *Onobrychis* species have been subjected to chemical investigations, among which most phytochemical studies were dedicated to *O. viciifolia*. This plant contained condensed tannins, cinnamic acids, flavonoids, isoflavonoids, and acylated flavonol glycosides [12,13]. The aerial parts of *O. galegifolia* Boiss., *O. albiflora* Hub.–Mor., *O. tournefortii* (Willd.) Desv., and *O. argyrea* Boiss. subsp. *argyrea* Boiss. exhibited antidiabetic activity in alloxan-induced diabetic mice [14]. The extracts of flowers and roots of *O. armena* Boiss & Huet displayed antioxidant and antimicrobial activity against some human and fish pathogens [15]. Strong antioxidant activity was also exhibited by the methanol extracts of *O. sosnowiec, O. viciifolia*, and *O. melanotricha* [16], while the aerial parts of *O. oxyodonta* Boiss. showed moderate antibacterial and antitumor properties [17]. Several prenylated polyphenols, including isoflavone genistein derivatives, flavanone naringenin derivatives, and dihydro-stilbenes exhibiting a remarkable wound healing activity, were isolated from the crude acetone extract of leaves and flowers of *O. carduchorum* collected in Iraq [18].

A few investigations have been carried out on non-volatile metabolites isolated from crude extracts of *Onobrychis* species; by contrast, little is known about the composition of volatile fractions. To our knowledge, only the essential oil from *O. armena* has been analyzed so far [19]. Thus, we found it interesting to study the volatile fractions isolated from *O. carduchorum*. In fact, there is an increasing interest in the analysis of essential oils, due to the characteristic chemical composition and the wide variety of biological activities, which include antibacterial, antiviral, antioxidant, and anticancer properties. Not less important are the industrial applications of essential oils [20,21,22,23,24,25,26,27,28]. In this context, the study of essential oils isolated from species of little-investigated genera, such as *Onobrychis*, is attracting worldwide attention nowadays. Moreover, we considered that some volatile components of *O. carduchorum* may contribute to the wound healing effects of the plant, as they can be released by rubbing the leaves on skin lesions.

**Figure 1:**
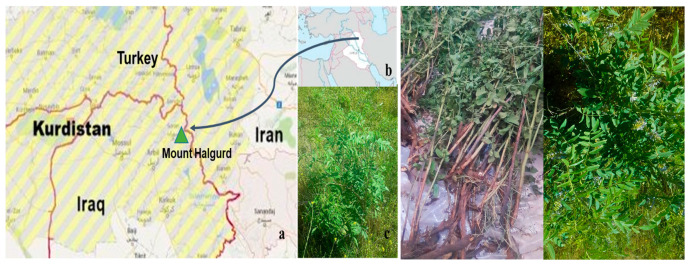
(**a**) Geographical position of Mount Halgurd in Iraqi Kurdistan; (**b**) position of the region in the Middle East; (**c**) *Onobrychis carduchorum* (photos taken by one of the authors, H.I.M.A.).

In this paper, we report, for the first time, the chemical–physical properties and the composition of the essential oils isolated from the aerial parts (OCA) and from the roots (OCR) of *O. carduchorum* (Figure 1c) collected on Mount Halgurd in the province of Erbil of the Kurdistan Autonomous Region, Iraq (Figure 1a,b). Moreover, a preliminary evaluation of the antiradical and antiproliferative effects of the two oils is also described. In fact, some essential oils (EOs) have been labeled as promising anticancer agents and are currently being investigated for their cytotoxic and antiproliferative activities in cancer cell lines or experimental animals [24,26,27,28]. Different mechanisms have been reported for the cytotoxic effects of EOs or their constituents. These include induction of cell death by apoptosis and/or necrosis; antimutagenic, antiproliferative, and antioxidant properties; cell cycle arrest; and loss of key organelle functions [27].

## 2. Results and Discussion

### 2.1. Physical Properties of the Essential Oils

The essential oils from aerial parts (OCA) and roots (OCR) of *O. carduchorum* had a yellowish color and a pleasant aromatic odor. The oils’ isolated yields were 0.15 ± 0.01 and 0.06 ± 0.01% (*w*/*w*), respectively, on air-dried plant material; the specific optical rotations of OCA and OCR oils were ${\left[\alpha \right]}_{D}^{20}$ +1.44 and ${\left[\alpha \right]}_{D}^{20}$ +2.73 (c = 0.1, CH_2_Cl_2_), respectively; the relative densities were 0.81 ± 0.02 and 0.83 ± 0.03 g/L, respectively.

### 2.2. Chemical Composition of the Essential Oils

The compounds identified in the OCA and OCR oils, with the corresponding percentages, are listed in Table 1, together with the corresponding retention times (RTs) and determined linear retention indices (LRI_exp_s).

The terpenoids identified in the two oils have been grouped in families in Table 2, while the other identified compounds have been divided according to chemical class. Compounds occurring in traces were not considered.

### 2.3. Antiproliferative Activity

The in vitro growth-inhibitory effects of OCA and OCR oils on T-47D human breast cancer cells, MDA-MB-453 human breast adenocarcinoma, BG-1 human ovarian carcinoma, A549 human adenocarcinoma alveolar basal cells, and two normal cell lines, namely, human embryonic kidney cells (HEK-293) and human fibroblast cells (HFF-1), were evaluated by the MTS assay [36]. The well-known anticancer drug cisplatin (*cis*-diamminedichloroplatinum (II)) was the reference compound. The results are reported in Table 3. The IC_50_ values ± SDs (μg/mL) were calculated as the means of three replicates. The goodness-of-fit of the probit regression model was assessed using the Pearson χ^2^ test.

### 2.4. Antiradical Activities

**Figure 2:**
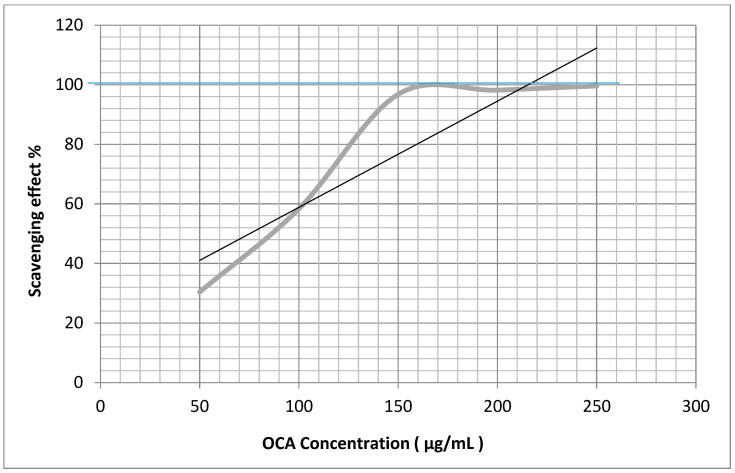
% DPPH radical scavenging effect vs. concentration (μg/mL) of the essential oil isolated from the aerial parts of *O. carduchorum* (OCA).

**Figure 3:**
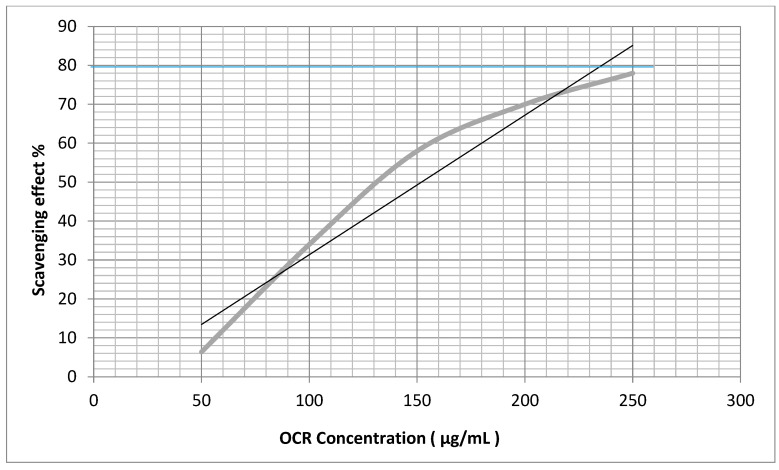
% DPPH radical scavenging effect vs. concentration (μg/mL) of the essential oil isolated from the roots of *O. carduchorum* (OCR).

The antioxidant and antiradical activities of the OCA and OCR oils were evaluated by measuring the scavenging properties towards H_2_O_2_ and the stable DPPH (2,2-diphenyl-1-picrylhydrazyl) hydroxyl radical (Figure 2 and Figure 3), using ascorbic acid as a positive control. The EC_50_ values ± SDs (μg/mL) are reported in Table 4 and were calculated as the means of three replicates.

## 3. Discussion

**Figure 4:**
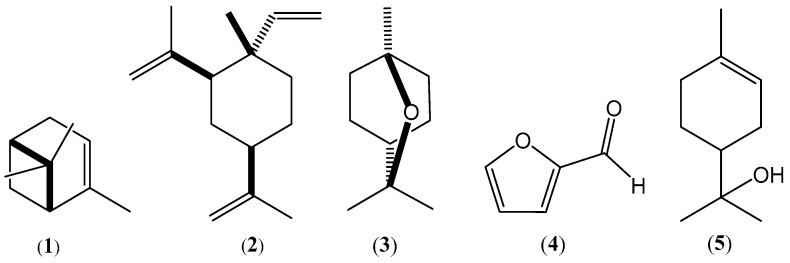
Chemical structures of the main components of the essential oils hydrodistilled from the aerial parts and roots of *O. carduchorum*: α-pinene (**1**), β-elemene (**2**), 1,8-cineole (**3**), furfural (**4**), and α-terpineol (**5**).

The most abundant constituents of the OCA oil were monoterpene hydrocarbons, followed by oxygenated monoterpenoids, while oxygenated monoterpenoids were largely predominant in the OCR oil. α-Pinene (**1**), β-elemene (**2**), 1,8-cineole (**3**), furfural (**4**) (Figure 4), terpineol-4-ol, and limonene were, in that order, the most abundant constituents of the OCA oil, accounting for about 70% of the oil composition (Table 1). On the other hand, 1,8-cineole (**3**), furfural (**4**), β-elemene (**2**), α-terpineol (**5**) (Figure 4), and linalool were, in that order, the most abundant constituents of the OCR oil, making up about 60% of the oil composition (Table 1).

Our findings indicated that the compositions of the two essential oils isolated from *O. carduchorum* aerial parts and roots differed greatly from that of the essential oil isolated from *Onobrychis armena*, which was rich in high-molecular-weight hydrocarbons and fatty acids [19]. Thus, the occurrence of α-pinene (**1**), β-elemene (**2**), 1,8-cineole (**3**), and furfural (**4**) markedly characterized the essential oils from *O. carduchorum* collected in Iraqi Kurdistan, although the percentages of these constituents varied between the two oils. Such variations were likely the basis of the small differences between the oils’ biological activities and the odors. Thus, the main contributor to the smell of the OCA oil was possibly the most abundant component, α-pinene (23.11 ± 0.1%), which has a characteristic intense turpentine scent. By contrast, the smell of the OCR oil was probably due to a combination of the smells of different components.

On the other hand, the presence of large amounts of β-elemene (**2**) in the two oils suggested the existence of a chemotype of *O. carduchorum* grown in Iraqi Kurdistan.

Regarding the antiproliferative activity of the oils against cancer cells in the MTS assay [36] (Table 3), the OCA oil exhibited high activity towards A549, BG-1, and T-47D human cancer cell lines, although the effects were significantly weaker than cisplatin. Of note, the minimal activity displayed against MDA-MB-453 cells appeared to exclude a non-specific antiproliferative activity against cancer cells. On the other hand, the OCR oil possessed significant antiproliferative activity against T-47D and MDA-MB-453 cell lines. In contrast, the efficacy of OCA and OCR oils towards two normal human cell lines, HEK-293 and HFF-1, was weaker than against cancer cells. Taken all together, the results of the MTS assay (Table 3) suggested that the two oils had a specific antiproliferative activity against certain cancer cells.

We considered the toxicity of the two oils quite interesting. In fact, based on the criteria of the U.S. National Cancer Institute (NCI), the cytotoxicity of essential oils is classified as follows: IC_50_ ≤ 20 µg/mL = highly cytotoxic, IC_50_ range between 21 and 200 µg/mL = moderately cytotoxic, IC_50_ range between 201 and 500 µg/mL = weakly cytotoxic, IC_50_ > 501 µg/mL = non-cytotoxic [37]. The cytotoxicity of the two oils likely resulted from a complex interaction of various oil components, acting with cellular structures and processes both alone and in synergy [27,28]. Moreover, some oil constituents could reduce the concentration of active components or have antagonist effects. The terpenoids limonene, 1,8-cineole (**3**), linalool, and the powerful antitumor agent β-elemene (**2**) [38,39], which was highly abundant (10–17%) in both oils, were possibly the main constituents responsible for the observed antiproliferative effects. It is remarkable that the sesquiterpene β-elemene (**2**) and various derivatives inhibited tumor cell growth even in vivo and demonstrated significant efficacy in clinical trials with cancer patients [40].

The oxidation process is a chemical reaction that produces free radicals, leading to chain reactions. A chain-breaking antiradical/antioxidant agent has the capacity to scavenge free radicals, typically by donating a hydrogen atom and forming a relatively stable radical which is unable to propagate the chain reaction. Alternately, the antioxidant undergoes autoxidation characterized by a very fast termination process [41]. It must be noted that the terms antiradical and antioxidant are often considered synonyms. However, strictly speaking, an antioxidant is an antiradical agent capable of quenching the radical species involved in oxidative chain carrying, that is, a peroxyl radical [42].

It has been proposed that antioxidants may play a role in the prevention and treatment of certain diseases, such as inflammation, aging, brain dysfunction, cancer, heart disease, arthritis, and the decline of the immune system. In fact, among the causes of the etiology and progression of these pathologies, evidence has indicated the adverse effects of an excess of free radicals and reactive oxygen species, such as H_2_O_2_, that react with biomacromolecules and cause severe damages to cells [42,43]. This pathological condition is called oxidative stress, which corresponds to a serious imbalance between the production of free radicals and the antioxidant inherent defense system of an organism [43]. In this situation, to prevent oxidation, the addition of either synthetic or natural antioxidants to fats, foods, and cosmetics is a common practice. Many essential oils have antioxidant properties, and their use as natural antioxidants is a field of growing interest because some synthetic antioxidants are now suspected to be potentially harmful to human health.

To obtain a preliminary indication of the antioxidant/antiradical properties of the OCA and OCR oils isolated from *O. cardochorum*, we used two standard tests (Table 4), although we were aware of the limited significance of such methods. In fact, according to some authors, the results would indicate a “radical trapping power” rather than true antioxidant activity [41]. The two oils showed comparable radical scavenging activities in the two tests, although they were less potent than ascorbic acid, which was used as a reference (Table 4). It is very difficult to attribute these effects to one or some active principles and to identify a specific pathway of molecular action because the oils contained mixtures of different components which can act with different mechanisms, both individually and synergistically [24,41]. Monoterpene alcohols, such as linalool; ketones; aldehydes; hydrocarbons, such as limonene; and ethers, such as 1,8-cineole (**3**), are probably the main contributors to the free radical scavenging activity of the OCA and OCR oils [21,44]; however, the activity of the strong antioxidant phenols cresol and carvacrol, although minor components of the oils, may not be negligible.

## 4. Materials and Methods

### 4.1. Plant Material

Aerial parts (flowers, leaves, and stems) and roots of *Onobrychis carduchorum* were collected in April 2022 at an altitude of 2100 m a.s.l. on Mount Mountain, Choman District, in Erbil province, Kurdistan Autonomous Region of Iraq. The plant was identified by A. Al-Khayyat, Professor of Botany at the Salahaddin University in Erbil; a voucher specimen has been deposited at the Herbarium of the Salahaddin University with the accession number 7235. Freshly cut aerial parts and roots were air-dried for a few hours at room temperature in the shade with an unforced ventilation.

### 4.2. Isolation of Volatile Fractions

Air-dried aerial parts (100 g) and roots (150 g) of *O. carduchorum* were separately hydrodistilled for approximately 5 h using a conventional Clevenger-type apparatus; this procedure was performed in triplicate for each sample. After decantation, each layer of oil was separated from the aqueous phase using a pipette; subsequently, the oils were dried over anhydrous sodium sulphate, filtered, and kept in the dark at −20 °C until analysis. The density, optical rotation, and yield of the oils were determined by standard methods [45].

### 4.3. GC-MS Analysis

GC-MS analyses were performed on a Thermo Scientific Focus GC instrument coupled to a DSQ mass spectrometer detector operating in an electron-impact (EI) mode with a voltage of ionization of 70 eV. A slightly polar Agilent J&W HP-5 fused silica capillary column (5% phenylmethylpolysiloxane, 30 m × 0.25 mm i.d.; 0.25 µm film thickness; Agilent Technologies Italia S.p.A, Cernusco sul Naviglio, MI, Italy) was used. The carrier gas was He at a constant flow rate of 1.0 mL/min. Each essential oil was dissolved in CH_2_Cl_2_ to a dilution of 1 mg/10 mL, and 1 µL of the solution was injected in split mode (20:1). The oven temperature was maintained at 60 °C for 1 min, then it was increased to 260 °C at a rate of 5 °C/min and held at 260 °C for 5 min. Acquisition mass range: *m*/*z* 41–350 amu; injector and transfer-line temperatures: 250 °C. Data were analyzed with MSD ChemStation software.

### 4.4. GC-FID Analysis

GC separations were carried out on a Perkin Elmer GC 2400 instrument (Perkin Elmer Italia SPA, Milan, Italy). A slightly polar Agilent J&W HP-5 fused silica capillary column (5% phenylmethylpolysiloxane, 25 m × 0.32 mm i.d.; 1.05 µm film thickness; Agilent Technologies Italia S.p.A, Cernusco sul Naviglio, MI, Italy) was used. The carrier gas was He at a constant flow rate of 1.0 mL/min. The injector temperature was 250 °C, and the FID detector was set at 260 °C; the oven temperature was maintained at 60 °C for 3 min, then it was increased to 260 °C at a rate of 5 °C/min and held at 260 °C for 1 min. A quantity of 1 µL of each essential oil solution (1 mg/10 mL in CH_2_Cl_2_) was manually injected in split mode (27:1). The relative amount of each oil component (Table 1) was calculated as the percent of the corresponding FID peak area with respect to the total area of peaks, without applying a correcting response factor. Mean % abundances and standard deviations were determined from the results of three replicates for each oil. Data were collected with HP3398A GC Chemstation software (Hewlett–Packard, Rev. A.01.01).

### 4.5. Identification of the Essential Oil Components

Each chemical component of the two oils (Table 1) was identified by comparing the corresponding mass spectrum with the spectra contained in the Adams [33] and NIST 08 [34] libraries, as well as by comparing the calculated linear retention index (LRI_exp_) with the literature [30,31,32,33,34,35]. Each LRI was calculated relative to a homologous series of standard *n*-alkanes (Sigma-Aldrich, Milan, Italy, No. CE 203-777-6), from *n*-octane (C_8_) to *n*-tricosane (C_23_), according to the van Den Dool and Kratz method [29]. The identification of most oil components was confirmed by coelution with authentic standards (Sigma-Aldrich, Milan, Italy).

### 4.6. Determination of the Antiproliferative Effects of the Oils (MTS Assay)

The MTS assay for evaluating the in vitro antiproliferative effects of a sample is based on the reduction of the yellow-colored MTS inner salt [3-(4,5-dimethylthiazol-2-yl)-5-(3-carboxymethoxyphenyl)-2-(4-sulfophenyl)-2H-tetrazolium] by NAD(P)H-dependent dehydrogenase enzymes in metabolically active cells to a purple formazan salt that is soluble in cell culture media [36]. The formazan salt has an absorbance maximum near 490 nm. When cells die, they lose the ability to convert MTS into formazan; color change thus serves as a convenient marker of only the viable (living) cells, and the measure of the absorbance can be directly related to the number of viable cells.

#### 4.6.1. Cell Cultures

Four human cancer cell lines, breast cancer T-47D, apocrine breast cancer MDA-MB-453, lung cancer A549, and ovarian adenocarcinoma BG-1, and two normal cell lines, namely, human embryonic kidney cells (HEK-293) and human fibroblast cells (HFF-1), obtained from the American Type Culture Collection (ATCC) and Sigma Aldrich, were used in the test. The cells were cultured in RPMI-1640 or DMEM/F-12 medium (Euroclone, S.p.A., Milan, Italy) supplemented with 10% fetal bovine serum (FBS), 100 μg/mL penicillin/streptomycin, and 2 mM L-glutamine (Life Technologies, Milan, Italy) at 37 °C under a humidified atmosphere in the presence of 5% CO_2_, changing the liquid growth medium whenever needed. When a cell culture reached 80% confluence, a small amount of trypsin was added to the medium to separate the cells from the flask; after 3 min of incubation at 37 °C, 1 mL of FBS was added to stop the action of trypsin and to avoid degradation of cell membranes. Subsequently, the cell-containing medium was transferred into a cell strainer and centrifuged (ALC 4232 Centrifuge) at 1000 rpm for 10 min. The resulting pellet was resuspended in the growth medium (1 mL), and the cells were separated using an automatic pipette and counted using a counting chamber and trypan blue as dye. Quadruple cell samples were grown in 96-well flat-bottom microtiter plates (Cellstar, Greiner bio-one) at a density of 5 × 10^5^ cells/mL of growth medium in each well. After 2 h of incubation, the medium was replaced with 100 µL of test medium (RPMI 1640, added with 0.005% L-glutamine, penicillin, and streptomycin), and the microplates were left in the incubator for an additional 24 h.

#### 4.6.2. MTS Assay of the OCA and OCR Oils

To determine the antiproliferative effects, six solutions of the OCA and OCR oils and cisplatin were prepared separately. Their concentrations ranged from 0.5 to 50 µg/mL in dimethyl sulfoxide (DMSO). The solvent DMSO was also tested for possible inhibitory effect by adjusting its concentration to be the same as the working concentration. The medium in the wells containing cultured cells was replaced with a solution (100 µL) of increasing sample concentration. Three replicates were performed for each sample dilution. The microplates were then incubated for 24 h in a humidified atmosphere of 5% CO_2_ at 37 °C; subsequently, the sample-containing medium was replaced with fresh test medium (100 µL) and 20 µL of MTS tetrazolium reagent (CellTiter 96^®^–AQueous One Solution Cell Proliferation Assay, Promega Italia S.R.L., Milan, Italy) was added. The plates were incubated for 2 h at 37 °C and the extent of MTS reduction was measured spectrophotometrically at 490 nm using a plate reader (BioRAD Model 550 Microplate Reader). Experiments were conducted in triplicate at room temperature. Cytotoxicity was expressed as the concentration of compound inhibiting cell growth by 50% (IC_50_). The IC_50_ values (µg/mL) (Table 3) were calculated by probit analysis (*p* < 0.05, χ^2^ test) with the GraphPad Prism 4 computer program (GraphPad Software, S. Diego, CA, USA).

### 4.7. Evaluation of Antiradical and Antioxidant Activities

The in vitro antiradical and antioxidant potential of the OCA and OCR oils were evaluated by the 1,1-diphenyl-2-picrylhydrazyl radical (DPPH) scavenging test [46,47] and by the H_2_O_2_ scavenging assay [48].

#### 4.7.1. DPPH Test

The method described by Vani [47] was used with a few modifications. Eight solutions (3 mL) of ascorbic acid or an oil at 5, 10, 25, 50, 100, 150, 200, and 300 µg/mL concentrations in MeOH-H_2_O, 9:1, were added individually to a fresh solution of DPPH in MeOH (1 mL, 0.3 mM). Subsequently, each mixture was shaken vigorously and incubated for 30 min at 22 °C in the dark until a deep violet color and a stable absorbance value (A) at 517 nm was observed. The absorbance was measured against the blank using a UV-Visible spectrophotometer (Lambda 25 UV/Vis spectrometer N.3903, Perkin Elmer instruments, Waltham, MA, USA). A lower absorbance of the reaction mixture indicated a higher free radical (DPPH^.^) scavenging (FRS) activity. The DPPH solution (1 mL) added by 10% aqueous MeOH (3 mL) was used as the control. The FRS% was calculated using the formula: [1 − (A_sample_/A_control_)] × 100, where A_control_ is the absorbance of the control at *t* = 0 min and A_sample_ is the absorbance at *t* = 30 min in the presence of the oil or ascorbic acid (Sigma-Aldrich, Milan, Italy), which was used as the standard. The curve of the % scavenging activity against the concentration was plotted for each sample (Figure 3 and Figure 4) using the MS Excel-based program to calculate the value of the EC_50_, i.e., the concentration (µg/mL) of the sample required to scavenge 50% of the DPPH concentration. Each analysis was carried out in triplicate at room temperature. The lower the EC_50_ value, the higher the antiradical activity of the sample.

#### 4.7.2. H_2_O_2_ Test

The method described in reference [48] was used with a few adjustments. After preliminary experiments, methanol solutions of the tested oil or ascorbic acid (2 mL; 100, 250, 500, and 1000 μg/mL) were added to a fresh hydrogen peroxide solution (0.6 mL, 40 mM) in phosphate buffer (pH 7.4). Ten minutes later, the absorbance of hydrogen peroxide at 230 nm was determined against a blank solution containing phosphate buffer without H_2_O_2_. The buffered H_2_O_2_ solution (0.6 mL, 40 mM) added by MeOH (2 mL) was used as the control. The H_2_O_2_ scavenging activity (%) was then calculated using the following equation: H_2_O_2_ scavenging effect % = [(A_control_ − A_sample_)/A_control_)] × 100, where A_control_ is the absorbance of the control and A_sample_ is the absorbance in the presence of the oil or ascorbic acid (Sigma-Aldrich, Milan, Italy), which was used as the standard. The curve of the % scavenging activity against the concentration was plotted for each sample using the MS Excel-based program to calculate the EC_50_ value, i.e., the concentration (µg/mL) of the sample required to scavenge 50% of the H_2_O_2_ concentration. Each analysis was carried out in triplicate at room temperature. The lower the EC_50_ value, the higher the H_2_O_2_ scavenging activity of the sample.

### 4.8. Statistical Analysis

All statistical data shown were expressed as means ± standard deviations (SDs) of three independent experiments (*n* = 3) and were calculated by probit analysis. To determine the half maximal inhibitory concentration (IC_50_/EC_50_) values, we employed probit analysis for analyzing dose–response data. The observed responses were transformed into probits, and a regression analysis was performed to estimate the IC_50_/EC_50_ values and the slopes of the dose–response curves. The goodness-of-fit of the probit regression model was assessed using the Pearson χ^2^ test. The one-way analysis of variance (ANOVA) test was used to compare the efficacies of compounds. The software GraphPad Prism 4 (GraphPad Software Inc., San Diego, CA, USA) was used for the analyses. The probability value of (* *p* < 0.05) was considered to denote a statistically significance difference.

## 5. Conclusions

The first determination of the chemical compositions of the essential oils hydrodistilled from the aerial parts and roots of *Onobrychis carduchorum* contributes to the still poor phytochemical knowledge of the genus *Onobrychis*. Moreover, the two oils showed significant antioxidant and radical quenching potential in vitro and interesting specific antiproliferative effects against a few human cancer cell lines in the MTS assay. However, before considering these oils as potential targets for the research of new antineoplastic agents, more investigations are needed, such as evaluation of cell morphology, exploration of the mechanisms of action, and in vivo studies aimed at defining the pharmacokinetic profile, safety, and toxicity of the oils.

As a conclusive remark, the wide range of biological activities determined for the essential oils from *O. carduchorum*, together with the properties previously found for the non-volatile metabolites, gives scientific support to the use of the plant in traditional Kurdish medicine.

## Figures and Tables

**Table 1:** Chemical composition of the essential oils from aerial parts and roots of *O. carduchorum*.

No. of Identified Compound	Compound Name ^a^	RT ^b^	LRI_exp_^c^	LRI_lit_^d^	% MS Match ^e^	% in the OCA Oil ^f^ ± SD	% in the OCR Oil ^g^ ± SD
1	*n*-Octane ^h^	2.12	801	800	94	2.26 ± 0.01	0.32 ± 0.02
2	Hexanal ^h^	3.88	803	801	93	0.13 ± 0.02	2.22 ± 0.11
3	**Furfural** ^h^	3.98	825	828	93	7.91 ± 0.06	10.44 ± 0.08
4	2-Methylbutanoic acid ^h^	4.11	831	832	87	0.77 ± 0.03	0.34 ± 0.03
5	Ethyl isovalerate ^h^	4.31	845	849	89	Tr	0.55 ± 0.02
6	(2*E*)-Hexenol ^h^	5.17	855	854	90	Tr	0.05 ± 0.01
7	1-Hexanol ^h^	5.53	862	863	89	0.83 ± 0.02	0.24 ± 0.02
8	Heptanal ^h^	6.34	903	901	89	2.01 ± 0.01	0.83 ± 0.03
9	Methional ^h^	7.03	911	909	90	1.45 ± 0.03	Tr
10	2-Acetylfuran ^h^	7.21	915	909	90	Tr	2.14 ± 0.06
11	**α-Pinene** ^h^	7.93	935	932	88	23.11 ± 0.06	4.76 ± 0.12
12	3-Methylcyclohexanone ^h^	7.98	946	945	78	0.77 ± 0.01	Tr
13	Benzaldehyde ^h^	8.01	959	952	95	0.32 ± 0.02	0.54 ± 0.04
14	β-Pinene ^h^	8.18	971	974	90	0.29 ± 0.02	4.37 ± 0.14
15	Isomaltol	8.26	985	980	94	0.07 ± 0.01	0.03 ± 0.01
16	1,4-Cineole ^h^	8.34	996	991	83	0.17 ± 0.01	Tr
	Unidentified	9.11	1006	-	-	Tr	0.43 ± 0.02
17	α-Terpinene ^h^	9.81	1018	1014	90	1.65 ± 0.04	Tr
18	Limonene ^h^	10.23	1022	1024	88	4.13 ± 0.06	2.89 ± 0.07
19	**1,8-Cineole** ^h^	10.59	1024	1026	93	12.15 ± 0.05	15.79 ± 0.12
	Unidentified	11.22	1045	-	-	0.54 ± 0.03	0.67 ± 0.03
20	*m*-Cresol ^h^	11.39	1069	1072	74	0.54 ± 0.03	Tr
21	*cis*-Linalool oxide ^h^ (furanoic)	11.74	1078	1067	83	1.23 ± 0.04	1.33 ± 0.06
22	*trans*-Linalool oxide ^h^ (furanoic)	12.09	1081	1084	67	0.32 ± 0.02	Tr
	Unidentified	13.19	1089	-	-	0.42 ± 0.02	0.34 ± 0.03
23	Linalool ^h^	13.67	1100	1095	80	2.70 ± 0.04	7.45 ± 0.06
24	Nonanal ^h^	13.74	1105	1100	64	0.43 ± 0.02	Tr
25	Methyl octanoate ^h^	13.91	1130	1123	68	Tr	2.3 ± 0.07
26	1,4-Dimethoxybenzene ^h^	14.04	1163	1161	82	0.28 ± 0.04	0.07 ± 0.01
27	Octanoic acid ^h^	14.22	1171	1167	89	1.41 ± 0.01	2.45 ± 0.03
28	**Terpinen-4-ol** ^h^	14.39	1177	1174	90	6.32 ± 0.05	3.61 ± 0.07
29	**α-Terpineol** ^h^	15.57	1190	1186	81	1.32 ± 0.05	7.74 ± 0.07
30	Decanal ^h^	15.89	1205	1201	90	0.08 ± 0.02	0.05 ± 0.01
31	(*Z*)-Ocimenone	16.12	1231	1226	90	1.32 ± 0.04	Tr
	Unidentified	16.88	1243	-	-	Tr	0.67 ± 0.02
32	2-Phenylethyl acetate ^h^	17.65	1255	1260	79	1.30 ± 0.02	0.15 ± 0.01
33	Cinnamaldehyde ^h^	17.77	1258	1267	51	Tr	0.18 ± 0.02
34	Carvacrol ^h^	17.90	1297	1298	94	1.06 ± 0.03	6.41 ± 0.05
35	Undecanal ^h^	17.98	1299	1305	84	0.23 ± 0.04	0.03 ± 0.01
	Unidentified	18.94	1365	-	-	Tr	1.77 ± 0.09
36	**β-Elemene** ^h^	18.98	1385	1389	91	17.33 ± 0.07	10.14 ± 0.12
37	γ-Curcumene	19.09	1482	1481	95	0.03 ± 0.01	Tr
38	β-Ionone ^h^	19.33	1495	1486	82	1.35 ± 0.03	2.65 ± 0.03
	Unidentified	20.77	1520	-	-	Tr	0.65 ± 0.04
39	α-Cadinene	21.73	1540	1537	88	0.04 ± 0.01	0.11 ± 0.01
40	Zierone	22.45	1580	1574	83	0.11 ± 0.03	0.17 ± 0.03
	Unidentified	23.11	1583	-	-	0.12 ± 0.03	0.42 ± 0.02
41	Salvial-4(14)-en-1-one	24.25	1591	1594	81	0.03 ± 0.01	0.04 ± 0.01
42	Tetradecanoic acid	25.33	1777	1767	88	0.98 ± 0.05	0.55 ± 0.02
43	Octadecanoic acid ^h^	25.97	2165	2169	95	2.15 ± 0.06	2.39 ± 0.07

^a^ Compounds are listed in order of their elution from a HP-5 column; the names of major compounds in both oils are bolded. ^b^ Compound retention time (min) on a HP-5 column. ^c^ Linear retention index on an HP-5 column, experimentally determined using a standard homologous series (C_8_–C_23_) of *n*-alkanes [29]. ^d^ Linear retention index taken from the literature [30,31,32,33,34,35] for a slightly polar column. ^e^ % match of the experimental mass spectrum with the literature [33,34]. Identical mass spectra would produce a match of 100%. ^f,g^ % content ± SD of each component (*n* = 3) in the corresponding oil, calculated from the corresponding peak area in the FID gas chromatogram. Tr = trace amount (mean value below 0.03%). ^h^ Coeluted with a standard.

**Table 2:** Chemical classes of the compounds identified in the OCA and OCR oils.

Chemical Classes of Identified Compounds (Total Number in the OCA + OCR Oils)	% in OCA	% in OCR
**Terpenoids:**		
Monoterpene hydrocarbons (4)	29.18	12.02
Oxygenated monoterpenoids (8)	25.53	35.92
Sesquiterpene hydrocarbons (3)	17.40	10.25
Oxygenated sesquiterpenoids (2)	0.14	0.21
**Others:**		
Hydrocarbons (1)	2.26	0.32
Aldehydes (9)	12.56	14.29
Ketones (4)	2.19	4.82
Alcohols (2)	0.83	0.29
Carboxylic acids (4)	5.31	5.73
Esters (3)	1.30	3.00
Miscellaneous aromatic derivatives (3)	1.88	6.48
**Total identified compounds (43)**	98.58	93.33

**Table 3:** In vitro antiproliferative activity (IC_50_ values ± SDs, µg/mL) of the essential oils hydrodistilled from aerial parts (OCA) and roots (OCR) of *O. carduchorum*.

Sample	T-47D ^a^	MDA-MB-453 ^b^	BG-1 ^c^	A549 ^d^	HEK-293 ^e^	HFF-1 ^f^
OCA	12.1 ± 0.11 *	>50	11.2 ± 0.2 *	10.2 ± 0.3 *	26.1 ± 0.21 *	>50
OCR	16.3 ± 0.15 *	14.5 ± 0.2 *	23.4 ± 0.6 *	16.4 ± 0.4 *	30.8 ± 0.1 *	32.5 ± 0.3 *
Cisplatin	5.10 ± 0.04	3.09 ± 0.06	3.69 ± 0.11	3.96 ± 0.08	7.3 ± 0.07	7.09 ± 0.11

^a^ Human breast cancer cell line. ^b^ Human breast cancer cell line. ^c^ Human ovarian carcinoma cell line. ^d^ Human adenocarcinoma alveolar basal epithelial cells. ^e^ Human embryonic kidney cells. ^f^ Human fibroblast cells (* *p* < 0.05).

**Table 4:** In vitro scavenging activity (EC_50_ ± SD, μg/mL) of the essential oils isolated from the aerial parts (OCA) and roots (OCR) of *O. carduchorum*.

Sample	DPPH	H_2_O_2_
OCA	79.8 ± 0.5 *	394.1 ± 0.2 *
OCR	153.3 ± 0.6 *	311.7 ± 0.5 *
Ascorbic acid	19.84 ± 0.12	36.51 ± 0.10

* *p* < 0.05.

## Data Availability

Not applicable.
